# Resurgent Sodium Current in Neurons of the Cerebral Cortex

**DOI:** 10.3389/fncel.2021.760610

**Published:** 2021-10-01

**Authors:** Giulia Quattrocolo, Keagan Dunville, Maximiliano José Nigro

**Affiliations:** Center for Neural Computation, Egil and Pauline Braathen and Fred Kavli Center for Cortical Microcircuits, Kavli Institute for Systems Neuroscience, Norwegian University of Science and Technology, Trondheim, Norway

**Keywords:** resurgent sodium current, cortical neurons, hippocampus, sodium channels, neurophysiology

## Abstract

In the late ’90, Dr. Indira Raman, at the time a postdoctoral fellow with Dr. Bruce Bean, at Harvard University, identified a new type of sodium current, flowing through the channels that reopens when the membrane is repolarized. This current, called “resurgent Sodium current,” was originally identified in cerebellar Purkinje neurons and has now been confirmed in around 20 different neuronal types. Since moving to Northwestern University in 1999 to establish her own research group, Dr. Raman has dedicated great efforts in identifying the mechanisms supporting the resurgent Sodium current and how its biophysical properties shape the firing of the different cell types. Her work has impacted greatly the field of cellular neurophysiology, from basic research to translation neuroscience. In fact, alterations in the resurgent sodium currents have been observed in several neuropathologies, from Huntington’s disease to epilepsy. In this Perspective we will focus on the current knowledge on the expression and function of the resurgent Sodium current in neurons of the cerebral cortex and hippocampus. We will also briefly highlight the role of Dr. Raman’s as teacher and mentor, not only for her pupils, but for the whole scientific community.

## Introduction

Neuronal voltage-gated Sodium channels (VGSCs) are responsible for the large and transient inward current (I_NaT_) underlying the upstroke of the action potential (AP) (Hodgkin and Huxley, 1952a). After opening, VGSCs quickly inactivate and require strong membrane hyperpolarization to become available (Hodgkin and Huxley, 1952b). Many neurons express subthreshold non-inactivating Sodium currents: a persistent sodium current (I_NaP_) flowing through non-inactivated VGSCs, and resurgent Sodium current (I_NaR_) that activates upon membrane repolarization (Stafstrom et al., 1982; Crill, 1996; Raman and Bean, 1997). Subthreshold sodium currents can powerfully shape spike after-potentials and repetitive firing (Raman and Bean, 1997; Khaliq et al., 2003; Yamada-Hanff and Bean, 2013). I_NaR_ has first been described in isolated cerebellar Purkinje neurons, where it contributes to the generation of complex spikes and repetitive spontaneous firing (Raman and Bean, 1997; Khaliq et al., 2003). I_NaR_ has been identified in many different cell-types throughout the brain, with conserved biophysical properties (Lewis and Raman, 2014). Thanks to the elegant work of Dr. Raman and collaborators, I_NaR_, together with I_NaT_, is the best characterized component of the Sodium current. Indeed, Dr. Raman’s original description highlighted the role of I_NaR_ in bursting in Purkinje neurons and developed a model for its generation hypothesizing the existence of a blocking particle that enters the pore at open states and is released upon repolarization (Raman and Bean, 1997, 2001). Later, Dr. Raman led her laboratory on a quest to decipher the structural and molecular mechanisms of I_NaR_, discovering the interaction of alpha and beta subunits of VGSCs underlying open channel block and the identity of the blocking particle (Grieco et al., 2005; Aman and Raman, 2007, 2010; Aman et al., 2009; Bant and Raman, 2010; Lewis and Raman, 2011, 2013). Following its description in Purkinje cells, the discovery of I_NaR_ in other cell-types has propelled research on the molecular underpinnings and physiological role of I_NaR_ in the brainstem, basal ganglia and peripheral sensory neurons (Do and Bean, 2003; Cummins et al., 2005; Enomoto et al., 2006; Barbosa et al., 2015), as well as its involvement in disorders such as pain, long QT syndrome and epilepsy (Jarecki et al., 2010; Hargus et al., 2011). In this review we want to focus on I_NaR_ in neurons of the cerebral cortex, where the work of Dr. Raman inspired our own first steps in the field of neurophysiology. The authors started their journey in neuroscience by studying the expression of I_NaR_ in cortical neurons and were deeply inspired by the solidity and elegance of the experimental work of Dr. Raman. One of us (GQ) had the chance to attend Dr. Raman’s “Great experiments in Cellular Neurophysiology” course at Northwestern University, experiencing her outstanding teaching.

## Expression of I_NaR_ in Cortical and Hippocampal Neurons

Soon after Raman and Bean (1997) first described it in cerebellar Purkinje cells, the _INaR_ has been identified in several cell types of the cerebellum, brainstem, basal ganglia, and dorsal root ganglia (Lewis and Raman, 2014). Raman and Bean (1997) also described the absence of I_NaR_ in acutely isolated pyramidal neurons of the CA3 region. In the cerebral cortex, I_NaR_ was first reported in layer II of the rat perirhinal cortex (Castelli et al., 2007a). This first report was followed shortly by an examination of the expression of I_NaR_ across the hippocampus and parahippocampal region (Castelli et al., 2007b). In the perirhinal and entorhinal cortices 75–100% of excitatory neurons express I_NaR_ depending on layer localization. In these regions the resurgent conductance amounts to 1.5–3% of the conductance of the transient component. As opposed to other types of neurons, the channels responsible for the I_NaR_ in cortical neurons are enriched in the axon initial segment. Indeed, application of TTx to the axon initial segment of perirhinal neurons abolished I_NaR_ to a larger extent than when applied to the soma/proximal apical dendrite (Castelli et al., 2007a). Moreover, patch clamp recordings from acutely isolated cortical neurons rarely show I_NaR_ (Castelli et al., 2007a). In the parahippocampal region, I_NaR_ is expressed most prominently by layer II excitatory neurons of the medial entorhinal cortex (MEC) (Castelli et al., 2007b). In MEC layer II, I_NaR_ was found in all recorded neurons, with an amplitude representing 3.6% that of I_NaT_, the second largest among neurons of the cerebral cortex (Castelli et al., 2007b; Nigro et al., 2012). MEC layer II neurons express all three components of the Sodium current: I_NaT_, I_NaP_, and I_NaR_. The developmental expression of the I_NaR_ in MEC layer II follows a trajectory that is independent from that of the other components, increasing steadily in amplitude from postnatal day (P) 5 to P10 (Nigro et al., 2012). In parallel, the percent of neurons expressing I_NaR_ also increases in the same developmental window (Nigro et al., 2012).

In the hippocampus I_NaR_ is expressed in subpopulations of excitatory neurons in the dentate gyrus (60%), ventral CA1 (40%), and the majority of subicular neurons (Castelli et al., 2007b; Barker et al., 2017). Interestingly, I_NaR_ does not seem to be expressed by pyramidal neurons of the dorsal hippocampus or in CA3 pyramidal neurons recorded from brain slices (Castelli et al., 2007b). Future studies correlating I_NaR_ expression to transcriptomic cell types might shed light on the molecular identity of neurons expressing I_NaR_ and the molecular mechanisms underlying its expression in the cortex (see below).

## Contribution of I_NaR_ to Firing Properties of Cortical Neurons

Several neuron types expressing I_NaR_ show spontaneous firing with relatively high firing rates and bursting, e.g., Purkinje cells, subthalamic neurons, neurons of the cerebellar nuclei (Lewis and Raman, 2014). I_NaR_ endows these cell-types with the ability to produce repetitive spiking spontaneously (i.e., in absence of synaptic activity) at high frequencies by preventing fast inactivation through open channel block (Raman and Bean, 2001; Khaliq et al., 2003). At depolarized membrane potentials, the open channel block competes with fast inactivation, and during repolarization, the transition from open channel block to open states allows Sodium ions to flow and initiate a new cycle of spiking (Raman and Bean, 2001). Excitatory neurons of the cerebral cortex do not produce spontaneous repetitive firing, nor they reach high firing frequency, but fire trains of action potentials with different degrees of adaptation. I_NaR_ has been shown to contribute to repetitive firing in layer II pyramidal neurons of the rat perirhinal cortex (Castelli et al., 2007a). These neurons produce repetitive firing up to 30 Hz upon depolarizing current injection, a firing frequency much lower than other I_NaR_ expressing neurons outside the cerebral cortex. However, even at those firing frequencies I_NaR_ contributes to most of the Sodium current during the interspike interval promoting depolarization and repetitive firing (Castelli et al., 2007a).

By injecting AP waveforms (recorded in current clamp) in voltage clamp experiments, Raman and Bean (1997) demonstrated that I_NaR_ provides a major contribution to the generation of complex spikes in Purkinje neurons. Inspired by these original experiments, Alessi et al. (2016) tested the contribution of different ionic conductances to the generation of the depolarizing afterpotential (DAP) in MEC layer II stellate cells. The authors described two mechanisms generating DAPs in these cells acting at different membrane voltages. At hyperpolarized holding potentials T-type Calcium channels provide most of the depolarization following the fast afterhyperpolarization (fAHP). However, at holding voltages closer to the resting potential, subthreshold Sodium currents (I_NaP_ and I_NaR_) are the major contributors to the DAP (Alessi et al., 2016). During spatial navigation, MEC layer II stellate cells show a spatially modulated firing pattern characterized by regularly spaced firing fields arranged in a hexagonal matrix, as described for grid cells (Fyhn et al., 2004; Hafting et al., 2005; Domnisoru et al., 2013; Schmidt-Hieber and Hausser, 2013; Rowland et al., 2018). MEC stellate cells with grid firing patterns also show a higher probability of generating bursts of APs during navigation (Latuske et al., 2015; Bant et al., 2020). Interestingly, bursting probability and amplitude of I_NaR_ show a gradient along the dorso-ventral axis of the MEC that correlates with the gradient in spacing and field size of grid cells along the same axis (Bant et al., 2020). The authors observed that using bursts increased the performance of decoding the animal’s position during navigation as compared to isolated spikes. The higher information content of burst points to a cellular mechanism to maximize signal-to-noise ratio in dorsal MEC grid cells (Bant et al., 2020). We would like to highlight that these interesting results were obtained by Dr. Jason Bant, a previous student of Dr. Raman, teaming up with Dr. Lisa Giocomo, who pioneered the study of the topographic organization of biophysical properties in MEC.

## Molecular Mechanism of I_NaR_ in Cortical Neurons

**FIGURE 1:**
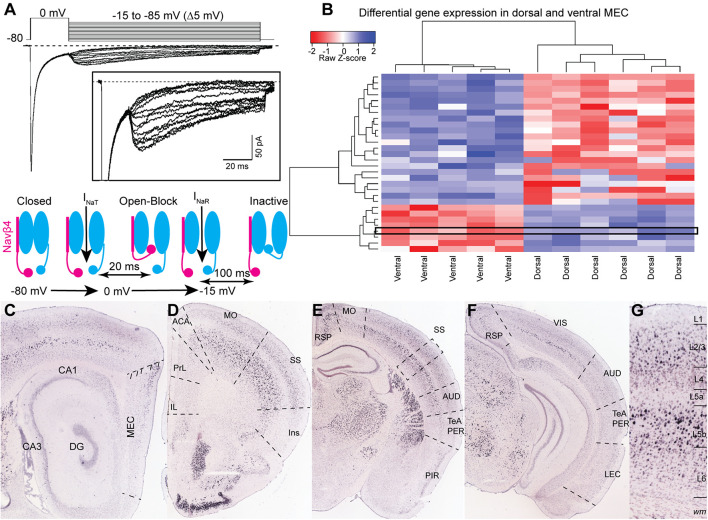
**(A)** Representative voltage-clamp recording of sodium currents in a layer II neuron in MEC. Sodium currents were evoked by the voltage steps shown in the upper panel. A 20 ms step to 0 mV evoked INaT and was followed by a family of repolarizing steps from –15 to –85 mV to elicit INaR. Insert shows an expanded view of the INaR. The schematic in the lower panel shows the sequence of events generating INaR in sodium channels associated with a Navβ4 subunit. **(B)** Differentially expressed gene analysis of data from Ramsden et al. (2015). Gene expression driven separation between ventral and dorsal medial entorhinal cortex from P60 mice. *Scn4b* is highlighted by a black rectangle and appears as one of 8 genes significantly enriched in the dorsal adult MEC. Heatmap displays 34 genes significantly up or downregulated in either ventral or dorsal MEC samples (*p*-val. < 0.05, log_2_FC threshold ± 1.5, Benjamini-Hochberg corrected; hierarchical clustering performed by pairwise-Spearman correlation matrix). **(C–G)** ISH experiments from the Allen Institute showing the expression of *scn4b* across the cerebral cortex. **(C)** *Scn4b* is expressed in the dorsal MEC. **(D–F)** Expression of *scn4b* in the isocortex decreases from rostral **(C)** to caudal **(F)** regions. **(G)** Magnification of the somatosensory (SS) area highlighted by a dotted box in **(C)**. CA, Cornu Ammonis; DG, dentate gyrus; MEC, medial entorhinal cortex; IL, infralimbic; PrL, prelimbic; ACA, anterior cingulate area; MO, motor area; SS, somatosensory area; Ins, insula; AUD, auditory area; TeA, temporal association area; PER, perirhinal cortex; PIR, piriform cortex; RSP, retrosplenial area; VIS, visual area; LEC, lateral entorhinal cortex. Image credit: For **(A)**: adapted from Nigro et al. (2012) with permission from Elsevier. For **(C–G)** Allen Institute. 2004 Allen Institute for Brain Science. Allen Mouse Brain Atlas. Available from: https://mouse.brain-map.org/.

Patch clamp experiments from Purkinje neurons obtained from Nav1.6 (*Scn8a*) null mice showed that this subunit contributes to most of the I_NaR_ in these neurons (Raman et al., 1997). However, the alpha subunit on its own cannot generate I_NaR_. Indeed Nav1.6 is also expressed in CA3 neurons that do not express I_NaR_ (Raman and Bean, 1997). The mechanism generating I_NaR_ involves a blocking particle that interacts with the alpha subunit (Raman and Bean, 2001). In a series of elegant experiments, Grieco et al. (2005) demonstrated that the blocking particle consists of the beta subunit Navβ4 (Grieco et al., 2005). Knockdown expression of the Navβ4 in cerebellar granule cells and peripheral sensory neurons strongly reduced I_NaR_, further highlighting the role of this beta subunit in generating I_NaR_ (Bant and Raman, 2010; Barbosa et al., 2015). In cortical neurons, Nav1.6 is expressed at high levels in the axon initial segment correlating with the subcellular expression of I_NaR_ in these neurons (Castelli et al., 2007a; Royeck et al., 2008; Buffington and Rasband, 2013). On the other hand, most of the cortical cells in which I_NaR_ has been observed express Navβ4 at very low levels (Buffington and Rasband, 2013) or completely lack *scn4b* expression (Yu et al., 2003; Lewis and Raman, 2014; Table 1). *In situ* hybridization (ISH) data from the Allen Brain Institute suggest a low level of expression in a subpopulation of layer II stellate cells in the dorsal MEC, which correlates with the dorso-ventral gradient of I_NaR_ in these neurons (Lein et al., 2007; Bant et al., 2020). In the hippocampus, *scn4b* is expressed in the dorsal CA1, where I_NaR_ is not expressed (Castelli et al., 2007b). The absence of I_NaR_ in CA1 pyramidal neurons might be explained by a slicing artifact by which the axon initial segment of CA1 neurons was lost. However, the recordings in CA1 were performed from coronal slices that preserve cellular integrity (Dougherty et al., 2012). Additionally, *scn4b* expression might not be sufficient for I_NaR_ expression in all cell types. A recent single cell transcriptomic analysis of the whole cortex and hippocampus by the Allen Brain Institute (Yao et al., 2021) showed that expression of *scn4b* is restricted to a handful of layer V neurons, none of whom are known to express I_NaR_. The discrepancy between ISH and single cell transcriptomic in MEC might be due to the sparseness of *scn4b* expressing neurons in this region (Figure 1). Additionally, this discrepancy may arise because of sample processing differences between ISH and single cell transcriptomics. In ISH, the tissue remains intact and preserves cytosolic compartments, like distal neuronal processes, that are otherwise destroyed in tissue homogenization for single cell RNA processing. Alternatively, single cell RNA sequencing intrinsically yields low rates of capture (Zheng et al., 2017) and read efficiency which must be accounted for computationally (Galfrè et al., 2021). Tissue homogenization is similar between bulk and single cell RNA sequencing and differences in technique should yield similar results. Bulk transcriptomic analysis comparing the dorsal and ventral MEC from adult mice in Ramsden et al. (2015), however, demonstrates that *scn4b* is significantly upregulated in the dorsal MEC at P60 (Figure 1B). Moreover, it is expressed at a level which drives distance-based clustering separation between the dorsal and ventral MEC (Ramsden et al., 2015). This discrepancy in the single cell dataset may not be related to weakness in gene detection or homogenization methods. Instead, gene expression differences in the dorsal-ventral axis in the adult single cell dataset may not have been considered and thus cells in ventral layer II may mask *scnb4* expression in dorsal layer II. Interestingly, a subgroup of pyramidal tract (PT) projecting layer V neurons showed a significant expression of *scn4b*, suggesting the possible expression of I_NaR_ in this type of cells. The expression of *scn4b* in layer V cortical neurons shows a gradient along the rostrocaudal axis of the telencephalon. Strong *scn4b* expression in layer V is evident in motor areas, sensory cortices, anterior cingulate and retrosplenial cortex. *Scn4b* seems to be absent in association areas, including the insula, parahippocampal area and medial prefrontal areas (Figures 1C–G). Future electrophysiological experiments might corroborate the presence of I_NaR_ in these cortical neurons. The molecular nature of the blocking particle in cortical neurons also remains enigmatic, particularly in the perirhinal cortex, ventral CA1 and dentate gyrus. Current approaches to characterize the transcriptomic and electrophysiological profile of neurons will shed light on the molecular underpinnings of I_NaR_ in cortical neurons (Cadwell et al., 2016).

**TABLE 1:** Cell-types where I_NaR_ expression and/or *scn4b* has been investigated.

Cell type	I_NaR_ expression	*scn4b* expression by ISH	References
CA3 pyramidal neuron	No	No	Raman and Bean, 1997; Yu et al., 2003 Allen Institute
Dorsal CA1 pyramidal neuron	No	Low	Yu et al., 2003 Allen Institute Castelli et al., 2007b
Ventral CA1 pyramidal neuron	35% of tested neurons	No	Yu et al., 2003 Allen Institute Castelli et al., 2007b
Dentate granule cells	60% of tested neurons	No	Yu et al., 2003 Allen Institute Castelli et al., 2007b
Subiculular pyramidal neurons	Most tested neurons	Yes	Allen Institute Barker et al., 2017
MEC LII stellate cells	Yes	Low in dorsal MEC (not detectable with single cell transcriptomics)	Allen Institute Castelli et al., 2007b
MEC LII pyramidal cells	Yes	Possibly in dorsal MEC	Allen Institute Castelli et al., 2007b
MEC LIII pyramidal cells	80% of tested neurons	No	Allen Institute Castelli et al., 2007b
MEC LV pyramidal neurons	70% of tested neurons	No	Allen Institute Castelli et al., 2007b
Perirhinal LII pyramidal neurons	90% of tested neurons	No	Allen Institute Castelli et al., 2007b
Perirhinal LV pyramidal neurons	80% of tested neurons	No	Allen Institute Castelli et al., 2007b
Neocortical LV pyramidal neurons	Unknown	Yes (detectable also with single cell transcriptomics)	Yu et al., 2003 Allen Institute

## I_Na*R*_ in Epilepsy

The contribution of I_NaR_ to repetitive spiking, DAP and burst generation suggests that it might play a role in disorders where neuronal excitability is altered, such as epilepsy. This component of the Sodium current is expressed in cortical areas that are strongly affected in temporal lobe epilepsy (TLE), such as MEC, perirhinal cortex, hippocampus and subiculum. I_NaR_ expressing MEC layer II neurons are spared in temporal lobe epilepsy, but they show an increased excitability (Bear et al., 1996). The increased excitability is in part due to a reorganization of the synaptic network (Kuman et al., 2007), however intrinsic mechanisms are also at play. Indeed, in the absence of synaptic inputs, layer II MEC neurons show a higher firing rate in response to current injection in a rat model of TLE (Hargus et al., 2011). These changes in intrinsic excitability correlate with an increased amplitude of all components of the Sodium current (Hargus et al., 2011). The molecular underpinnings of the increased excitability reside in an increased expression of the Nav1.6 subunit. Indeed, pharmacological blockade of Nav1.6 channels with 4,9-anidro-tetrodotoxin rescued the excitability of TLE MEC layer II neurons to levels like control neurons (Hargus et al., 2013). Moreover, similar alterations in intrinsic excitability and Sodium currents are recapitulated in mice carrying a mutated Nav1.6 isoform (Ottolini et al., 2017; Pan and Cummins, 2020). An increased excitability and augmented I_NaR_ and I_NaP_ have recently been described in human excitatory cortical neurons differentiated from pluripotent stem-cells obtained from patients affected by early infantile epileptic encephalopathy type 13 (*Scn8a*-related epilepsy) (Tidball et al., 2020).

## Discussion

In the current perspective we aimed at reviewing the state-of-the-art of the research on the I_NaR_ in neurons of the cerebral cortex. The expression of I_NaR_ has been reported in nine cortical cell-types, and we propose its expression in a population of layer V PT neurons based on their expression of Navβ4. The contribution of I_NaR_ to the firing behavior of cortical neurons has been well demonstrated in the perirhinal and entorhinal cortices. In these areas, I_NaR_ supports repetitive firing, DAPs and bursting. Bursting in LII MEC neurons has been proposed to maximize signal-to-noise ratio in grid cells and might represent a cellular mechanism for a reliable transmission of spatial information to the hippocampus (Bant et al., 2020).

The molecular mechanisms underlying I_NaR_ in cortical neurons are yet to be described. The molecular identity of the blocking particle in many cortical cell types remains unknown and future studies describing the correlation of trascriptomics and electrophysiology will allow to uncover potential candidates. These studies will also provide molecular targets for pharmacological treatments of epileptic encephalopaties involving I_NaR_.

With this perspective, we wished to emphasize the pivotal influence of Dr. Raman’s work on the mechanism of open-channel block, molecular identity of the blocking particle, and physiological role of I_NaR_ in cerebellar Purkinje neurons. Her findings sparked the quest for I_NaR_ in the cerebral cortex and provided the foundations for our current understanding of the role of I_NaR_ in the firing properties of cortical neurons.

In addition to her scientific contribution, Dr. Raman has also always been interested in forging new generations of scientists. Anyone who had the privilege of attending a class or a lecture given by Dr. Raman knows it will not be boring. Her scientific knowledge will engage you and her ability to introduce interesting anecdotes on the “behind the scenes” will enchant you. Her outstanding teaching abilities have been recognized by multiple awards received at Northwestern University, but her drive to good mentoring and good science has not stopped in Chicago. Dr. Raman has contributed to a series of feature articles titled “Living science” published in eLIFE from 2015 to 2019 (Raman, 2015a,b, 2016, 2017, 2019). In addition, her piece on good mentoring (Raman, 2014) faithfully describes the challenges faced by both the trainee and the advisor.

Dr. Raman contribution to science and her effort to make the scientific world a better place for young trainees and women has left a mark on the authors as well as, we are sure, on all the people that had trained with her and have known her.

## Data Availability Statement

Publicly available datasets were analyzed in this study. This data can be found here: https://mouse.brain-map.org/search/show?page_num=0&page_size=44&no_paging=false&exact_mat ch=true&search_term=Scn4b&search_type=gene. The bulk sequencing dataset can be found here: https://www. ebi.ac.uk/ena/browser/view/PRJNA267227?show=reads.

## Author Contributions

GQ and MN conceived the study and analyzed the data. KD analyzed the data. All authors wrote the manuscript.

## Conflict of Interest

The authors declare that the research was conducted in the absence of any commercial or financial relationships that could be construed as a potential conflict of interest.

## Publisher’s Note

All claims expressed in this article are solely those of the authors and do not necessarily represent those of their affiliated organizations, or those of the publisher, the editors and the reviewers. Any product that may be evaluated in this article, or claim that may be made by its manufacturer, is not guaranteed or endorsed by the publisher.
